# Mental health and resiliency following 44 months of terrorism: a survey of an Israeli national representative sample

**DOI:** 10.1186/1741-7015-4-21

**Published:** 2006-08-27

**Authors:** Avi Bleich, Marc Gelkopf, Yuval Melamed, Zahava Solomon

**Affiliations:** 1 Lev Hasharon Mental Health Center, PO Box 90000, Netanya 42100, Israel (affiliated to the Sackler Faculty of Medicine, Tel-Aviv University, Israel); 2 NATAL: The Israel Trauma Center for Victims of Terror and War, Israel; 3 Shappel School of Social Work and Adler Research Center, Tel Aviv University, Israel

## Abstract

**Background:**

Israeli citizens have been exposed to intense and ongoing terrorism since September 2000. We previously studied the mental health impact of terrorism on the Israeli population (Bleich et al., 2002), however the long-term impact of ongoing terrorism has not yet been examined. The present study evaluated the psychological sequelae of 44 months of terrorism in Israel, and sought to identify factors that may contribute to vulnerability and resilience.

**Methods:**

This was a telephone survey using strata sampling of 828 households, which reached a representative sample of 702 adult Israeli residents (84.8% contact rate). In total, 501 people (60.5%) agreed to participate. The methodology was similar to that of our previous study. Exposure to terrorism and other traumatic events, number of traumatic stress-related symptoms (TSRS), percentage of respondents with symptom criteria for post-traumatic stress disorder (PTSD), traumatic stress (TS) resiliency and feelings of depression, anxiety, optimism, sense of safety, and help-seeking were the main outcome measures.

**Results:**

In total, 56 participants (11.2%) were directly exposed to a terrorist incident, and 101 (20.2%) had family members or friends exposed. Respondents reported a mean ± SD of 5.0 ± 4.5 TSRS; 45 (9%) met symptom criteria for PTSD; and 72 (14.4%) were TS-resilient. There were 147 participants (29.5%) who felt depressed, 50 (10.4%) felt anxious, and almost half (235; 47%) felt life-threatening danger; 48 (9.7%) felt the need for professional help. Women and people of Arab ethnicity had more TSRS, more PTSD, and less TS resiliency. Injury following a life-threatening experience, a major stressful life event, and a major loss of income were associated with PTSD. Immigrant status, lower education, low sense of safety, low sense of social support, high societal distress, and injury following life-threatening experiences were associated with TSRS. TSRS did not increase with exposure severity. This study revealed less depression and functional impairment, similar rates of PTSD, increased help-seeking and poorer TSRS and TS resiliency than our initial study, 2 years previously.

**Discussion:**

The response of people in Israel to 4 years of terrorism is heterogeneous. Vulnerability factors change over time; Arab ethnicity, immigrant status and less education, not found to be risk factors in our previous study, were found in the present study to contribute to trauma-related distress. Prior experience of highly stressful events increases vulnerability to adverse psychological effects of terror.

## Background

Since late September 2000, when the Al-Aqsa Intifada (the second Palestinian uprising) erupted, Israel has experienced repeated deadly terror attacks, which have claimed large numbers of civilian casualties, disrupted daily life, and created an atmosphere of fear and insecurity. By May 2004, 1030 people had been killed, and 5788 injured in more than 13,000 terrorist attacks [1]. Approximately 0.1% of the population was injured or killed – an equivalent percentage in the USA would equate to some 295,000 people. The extensive, graphic, real-time, and repeated media coverage of the attacks has also contributed to the sense of a shared massive national crisis.

Given the recent rise in terror attacks worldwide, it is important to try to understand how people react to terror, the factors that foster vulnerability or promote resilience, and how society is affected over time.

Several studies have examined stress-related mental health symptoms and coping behaviors following terrorism. Studies of the impact of September 11 (the terrorist attacks in the USA in 2001) found that both people who experienced the attack directly [2] and those who experienced it indirectly, such as through the media [3,4], showed elevated levels of distress, lowered sense of security, and pathological reactions such as post-traumatic stress disorder (PTSD) and depression. Studies carried out in Spain [5], France [6,7], Ireland [8], Algeria [9], Sri Lanka [10], Australia [11], Guatemala [12], Japan [13], Britain [14] and Israel [15] similarly point to the psychological impact of exposure to terror.

On the whole, however, these studies do not address the impact of continuous terror on entire populations. The issues of whether responses to long-term, continuous terror differ from response to shorter episodes of terror and whether and how such terror affects vulnerability and resiliency have yet to be evaluated. Additional unresolved issues include whether there is a process of habituation that helps people learn to cope adaptively with terror or whether the continual stress of ongoing terrorism accumulates over time, and whether terror has different effects on different sectors of the population. In addition, the relation between ongoing terror and societal concerns has not yet been studied. Does the ongoing threat of continuous terror leave room for concern about other societal issues or does it so absorb people's thinking and emotional energy that it blocks out concern with such issues?

In the present study we also relate to the concept of resilience, defined by Bonano [16] (pp. 20–21) as 'the ability ... to maintain a relatively stable, healthy level of psychological and physical functioning' in the face of highly disruptive events. This concept is particularly important in view of findings following a range of traumatic events, which show that large percentages of people (40–78.2%) exposed to such events are either entirely or almost symptom-free [3,17-20].

The present study attempts to fill in some of the gaps. Conducted in May 2004, after 44 months of ongoing terror attacks in Israel, this is a follow-up of our previous study on Israelis' responses to terror [15]. The original study examined the psychological impact of terrorism on a representative sample of Israelis 19 months after the outbreak of the Al-Aqsa Intifada in October 2000 [15]. The present study was a telephone survey of a different sample of Israelis, and assessed a range of psychological responses among various sectors of the population: men and women, Jews and Arabs, religious and non-religious, Israeli-born and immigrants. The analyses focused on the correlates of and contributors to vulnerability and resilience. In addition to examining the role of individual factors (e.g., exposure, psychological features, demographic features, and prior life experiences), it also examined the possible role played by distress concerning other societal problems, a factor not considered in previous studies.

## Methods

### Sampling

The sample was obtained by a within-strata random-sampling method from a large database maintained by the Dahaf Polling Institute (Tel-Aviv, Israel). The database, pooling method and strata criteria have been described in our previous study [15].

The target population consisted of all adult Israeli residents aged 18 years or older. Accordingly, 828 households were telephoned. In total, 702 people were randomly reached by telephone (84.8% contact rate); of these 501 agreed to participate in the study, yielding a final participation rate of 60.5%, and a representative sample of the Israeli population with a maximum sampling error of 4.5%.

The participants' demographic characteristics are shown in Table 1. The sample consisted of 242 men (43.3%) and 259 women (51.7%), aged from 18 to 91 years (mean ± SD 44.8 ± 17.1). There were 430 Jews (85.8%) and 71 Israeli Arabs (14.2%). In the sample, 252 participants (56.3%) had received a year or more of post-high school education, 220 (43.9%) had completed high school, and 29 (5.8%) had attended only elementary high school. Of the Jewish Israelis, 39 (9.2%) reported that they were religious, 122 (28.6%) that they were traditional, 25 (5.9%) that they were ultra-orthodox, and 240 (56.3%) that they were atheist. Most of the sample lived in urban areas. Of the 430 Jews, 237 (55.4%) were born in Israel and 191 (44.6%) were immigrants; all the Arab participants were born in Israel. There were 189 (40.5%) participants who reported a net family income below the mean (about $2000 per month), 132 (28.3%) an average family income, and 146 (31.2%) a family income higher than the mean. The sample, which was roughly comparable to that in our previous study [15], was representative of the Israeli population, with no differences between the above distributions and data provided by the Israel Central Bureau of Statistics [21].

### Analysis of non-participants

Non-participants (*n *= 201) did not differ from participants (*n *= 501) on any demographic variable apart from having slightly lower income (mean ± SD 2.6 ± 1.3 vs. 2.8 ± 1.3); t_700 _= 2.0; *p *= 0.05) and, as in the previous study, being significantly younger (mean ± SD age, 42.0 ± 16.7 years) than participants (44.8 ± 17.1 years; t_700 _= 2.0; *p *= 0.05).

### Data collection

Interviews were conducted by telephone on 5 May 2004, using identical data collection procedures to those in our previous study [15]. Oral informed consent was obtained from participants at the beginning of the interview. The internal review board of Lev-Hasharon Mental Health Center approved the study.

### Instruments

The research instrument was a structured questionnaire consisting of 59 questions. Most of the questions were drawn from questionnaires used in the previous study to assess reactions to trauma [15]. The measures drawn from these questionnaires were level of exposure, PTSD symptoms; traumatic stress-related symptoms (TSRS), depression, sense of safety, optimism, and self-efficacy. In addition, we added questions tapping the respondents' anxiety, social support, previous experience with life-threatening events and physical injury ensuing from terrorist attacks, life events in the previous year, substantial loss of income at any time in the past, and experience of blatant ethnic discrimination at any time in the past. Finally, we asked whether a number of major societal problems caused the respondent personal distress. Except when otherwise indicated, the participants were asked to reply with respect to the time 'since the beginning of the Intifada'. Following a pilot study of 30 people (who were not included in the final sample), the questionnaire was modified to make it telephone-friendly. On average, the telephone survey took 12 minutes.

Exposure was assessed by questions asking the participants whether they had personally witnessed a terrorist attack and whether they had been injured by it, and whether friend(s) or family members had witnessed a terrorist attack and whether any of them had been injured or died in an attack. Based on the answers given, we divided the participants into nine exposure score (ES) groups: (i) no exposure, (ii) friend/family-only exposure, uninjured; (iii) friend/family-only exposure, injured; (iv) friend/family-only exposure, killed; (v) personal exposure only, uninjured; (vi) personal exposure and friend/family exposure, uninjured; (vii) personal exposure (uninjured) and friend/family injured or (viii) killed; and (ix) personal exposure with physical injury.

TSRS, PTSD, and traumatic stress (TS) resiliency were assessed using a modified 23-item version of the Stanford Acute Stress Reaction Questionnaire (SASRQ [22]). Subjects were asked to rate on a 5-point Likert scale (0 = disagree, 1 = agree somewhat, 2 = agree, 3 = strongly agree, 4 = totally agree) whether they had each stress symptom, and to report for how long: (i) 2 days or less, (ii) less than 1 month, (iii) longer than 1 month. A symptom was considered clinically relevant for TSRS and PTSD diagnosis if the individual at least 'agreed' (third choice out of 5). The scale showed a Cronbach's α of 0.88. Because not all respondents met the full DSM-IV criteria for PTSD (e.g. actual exposure to a traumatic event) and because our observations were made on the basis of screening instruments and not comprehensive clinical evaluations, the participants were not considered to have PTSD, but rather an aggregation of symptoms that met the criteria for PTSD. This included meeting the criteria for hyperarousal, re-experiencing, avoidance and functional problems, or a high level of distress.

Participants were considered TS resilient if they endorsed no TSRS item above 'a little'. To ensure that the measure did not assess denial, we compared, using data from our previous study, the use of denial as a coping strategy by TS resilient and non-resilient individuals. The comparison showed similar use by both groups (mean ± SD items endorsed: TS resilient (*n *= 119) 1.25 ± 0.7 vs. non-TS resilient (*n *= 390) 1.20 ± 0.7; t_508 _= 0.744; p = 0.5, NS). The same approach to assessing the absence of stress-related symptomatology was proposed by Galea et al [3].

Depression and anxiety were assessed via the statements: 'I feel depressed or gloomy' and 'I feel anxious or tense', rated on a 5-point Likert scale from 0 (very true) to 5 (not true at all) [4]. Personal optimism and optimism about the future of Israel were queried via two items adapted from the Children's Future Orientation Scale [23], rated on a 6-point Likert scale from 1 (very much agree) to 6 (do not agree at all). A response was considered positive if the participant indicated at least moderate agreement. Sense of safety was assessed by two statements developed for our previous study, which referred to a sense of threat to oneself and one's relatives, and which were rated on a 6-point Likert scale from 1 (strongly agree) to 6 (don't agree at all). Self-efficacy was assessed by the question, 'How well would you know what to do if you were caught in a terrorist attack?,' answered on a 6-point Likert scale from 0 (not at all) to 6 (very much). Responses of 3 and above were considered positive. The statistical reliability of these measures is presented in our previous study [15].

Sense of social support was assessed by one statement adapted from the Social Support Appraisal Scale designed by Vaux et al [24]: 'I can always rely on someone to help me when I'm in difficulty,' rated on a 6-point Likert scale from 1 (very true) to 6 (not true at all). Based on a telephone interview of a student sample, 2-week test-retest of this item was 0.92 (*n *= 30). A response was considered positive if the participant indicated at least moderate agreement [3].

Help-seeking was examined by asking participants two yes/no questions: (i) whether they were currently undergoing mental health treatment and (ii) whether they currently felt the need for such a treatment. To assess objective threat, we grouped together the participants who lived in high-risk areas (Jerusalem, Tel-Aviv, Netanya, Haifa, and West Bank settlements), where most of the suicide bombings had occurred, and compared their exposure with that of the participants who lived elsewhere. The comparison showed that participants residing in the high-risk areas (*n *= 129) had significantly higher exposure scores than those (*n *= 372) residing in lower-risk areas (mean ± SD 1.5 ± 2.1 versus 0.7 ± 1.6, respectively; t= 4.2; df = 499; *p *= 0.001).

Exposure to previous traumas was assessed by five yes/no questions: (i) life events in the previous year were tapped by asking respondents whether they had experienced a stressful or highly emotional event in the previous year; (ii) subjective economic hardship was assessed by asking whether they had ever suffered a major loss of income; (iii) subjective sense of ethnic discrimination was queried by asking whether they felt they had ever personally suffered blatant ethnic discrimination; (iv and v) previous exposure to life-threatening events was tapped by two questions: whether the respondents had ever been in a life-threatening situation due to accident, illness, terrorist attack or war; and whether they had sustained an injury of any consequence during these situations.

Distress related to major societal issues was assessed by asking the participants to rate how distressed they felt about 10 social problems: (i) terror, (ii) the economic situation, (iii) traffic accidents, (iv) ethnic discrimination, (v) the security situation, (vi) crime and violence, (vii) administration corruption, (viii) lack of leadership, (ix) the treatment of minorities (foreign workers, Israeli-Arabs, Bedouins etc.), and (x) the treatment of the Palestinians in the territories. This list represents the consensus of a panel of seven Israeli specialists in the behavioral sciences, who were asked to list all the 'social and national' issues about which Israelis get upset. Participants rated their distress about each issue on a 6-point Likert scale, from 1 (not at all) to 5 (very much). A societal distress score was computed by summing the ratings on all items except terror. In addition, respondents were asked to indicate which issue they regarded as the 'most distressing'. Based on a telephone interview of a student sample, 2-week test-retest of these items ranged from 0.75 to 0.92 (*n *= 30).

### Statistical analyses

Independent sample *t*-test, χ^2 ^tests, and Pearson's correlations were performed, followed by one-forward stepwise linear regressions for the continuous variables of TSR symptoms and two-forward conditional logistic regressions for categorical variables of symptom criteria of PTSD and TS resiliency. In each regression analysis, the significant predictors from nine groups of variables were tested for inclusion in the final models. Significance was set at 0.05 (two-tailed). Non-significant variables and variables that did not add to the predictive power of the model were removed from the regression to provide the most parsimonious model. No imputation of missing values was performed apart from the income variable score, which was replaced in the regression analyses with the sample mean (*n *= 34). Regression analysis omitting participants who did not complete the income variable did not significantly change any of the results. For other variables, cases were excluded from specific analysis when information was missing relative to the content analyzed. This did not substantially affect the size and the integrity of the sample. SPSS-PC version 11.5 (SPSS Inc, Chicago, IL, USA) was used for all analyses.

## Results

### Exposure, life events, life-threatening situations, injury following life-threatening situations, loss of income, and ethnic discrimination

The percentages of people exposed to terrorist attacks at the different degrees of exposure are presented in Table 2. More than one in 10 respondents (56; 11.2%) had witnessed a terrorist attack first-hand, and more than one in five (101; 20.2%) had friends or relatives who had witnessed an attack.

In total, 211 (42.3%) reported having experienced a non-terror highly stressful or emotional event in the previous year, while 179 (35.9%) reported having been in a life-threatening situation at some time in their lives. Of these 179, 36 (20.5%) reported having been seriously injured in that event. There were 175 people (35%) who reported having suffered a significant loss of income, and 70 participants (14%) felt they had been victims of ethnic discrimination. Further calculation indicated that women reported significantly more life events in the previous year than men (women 120/258 (56.9%) vs. men: 91/241 (37.8%); χ^2 ^= 3.9 (df = 1), *p *< 0.05), while men reported having experienced significantly more life-threatening events at some time in the past (men: 129/241 (53.5%) vs. women: 50/258 (19.4%); χ^2 ^= 63.2 (df = 1); *p *< 0.001).

### PTSD and TSR symptoms and TS resiliency

PTSD and TSR endorsements are presented in Table 3. With regard to PTSD symptoms, 180 (35.9%) of the participants endorsed at least one re-experiencing item (cluster B), 259 (51.7) at least one avoidance/numbing symptom (cluster C), 239 (47.7) at least one hyperarousal symptom (cluster D), 77 (15.4%) at least one of the two functional impairment items, and 221 (44.1%) the general distress item. More than a quarter (136; 27.1%) of the participants reported having at least one of the four dissociative symptoms. The mean ± SD number of dissociative symptoms endorsed was 0.4 ± 0.4). In total, 45 respondents (9.0%) met criteria for PTSD.

Regarding TSR symptoms, respondents endorsed a mean ± of 5.00 ± 4.5 symptoms out of the 23 queried. The mean ± SD TSRS intensity was 0.7 ± 0.6 (range 0–4). In total, 72 respondents (14.4%) reported no or minimal TSRS. These respondents were regarded as TS resilient.

### Feelings and attitudes

Table 4 presents trauma-related mental health attitudes and emotions. In total, 147 (29.5%) participants reported at least agreement with the statement 'I feel depressed and gloomy' and 50 (10.4%) stated that they agreed 'very much' or 'totally agreed'. A similar number, 145 (29%), at least agreed with the statement 'I feel anxious and tense'. Most respondents (409; 82%) reported that they felt optimistic about their personal future, and over half, 279 (56.5%) that they felt optimistic about the future of Israel. Almost half (235; 47%) felt that their lives were in danger, and over half (270; 54.1%) that the lives of family members or acquaintances were in danger. Around three-quarters (308; 76.6%) at least agreed with the statement that they would know what to do if they were caught in a terror attack. More than three-quarters (390; 78.2%) at least agreed with the statement that 'there will always be someone there to help me when I'm having difficulty'. Smaller proportions reported that they were receiving some sort of treatment for their mental health (18; 3.6%) and/or that they felt the need for such treatment (48; 9.7%).

### Distress about societal issues

As can be seen from Table 5, of all the issues that preoccupy Israelis, the economic situation was viewed as the most upsetting by the highest percentage (26.1%) of respondents. However, if we combine the 15.1% who named the security situation as the most upsetting with the 24.4% who named the terror attacks, then we see that 39.6% of the population viewed the violence and its implications as a more upsetting problem than any other in Israeli society. Very few respondents rated behavior towards Palestinians (3.5%), ethnic discrimination (2.5%) and behavior towards minorities (0.6%) as most upsetting.

### Objective threat/exposure and symptoms

Independent-samples *t *tests, χ^2 ^tests, and Pearson correlations showed no significant association between objective threat (high- vs. low-risk place of residence) or level of exposure and any of the independent variables. With 45 participants meeting symptom criteria for PTSD compared with 456 who did not meet the criteria, the analyses had a power of 44.7% to yield a statistically significant result. Neither was a significant difference found in the exposure to terror reported by the Jewish (*n *= 430) and Arab (*n *= 71) participants (mean ± SD 1.0 ± 1.7 vs. 0.9 ± 1.7 respectively; t_499 _= 0.4; *p *= 0.7).

### Demographic variables associated with symptoms

Meeting symptom criteria for PTSD was associated significantly with being female 12.7% (33/256) women vs. 4.5% (11/242) men; χ^2 ^= 10.5 (df = 1); *p *= 0.001), with being Arab (16.9% (12/71) Arabs vs. 7.4% (32/340) Jews; χ^2 ^= 6.8 (df = 1); *p *= 0.009), and with a somewhat lower level of education (participants who met symptom criteria for PTSD versus those who did not: mean ± SD 12.8 ± 3.1 vs. 13.8 ± 2.9 years of schooling; t_499 _= 2.2; *p *= 0.026). No other demographic feature was found to be significantly associated with meeting symptom criteria for PTSD.

In a similar vein, significantly more TSRS were found among women (women 6.3 ± 4.6 vs. men 3.6 ± 4.0; t_499 _= 7.1, *p *< 0.001), Arabs (Arabs 6.4 ± 4.6 vs. Jews 4.8 ± 4.5; t_499 _= 2.8; *p *= 0.005), and among those who were less educated (*r *= 0.16, *p *< 0.001). Significantly more TSRS were also found among participants born outside of Israel than among native-born Israelis (5.7 ± 4.6 vs. 4.6 ± 4.5; t_499 _= 2.5, p = 0.01), those who reported lower income (Pearson *r *= .15, *p *< 0.001), and those who were religiously observant Jews (religious Jews 5.3 ± 4.9 vs. non-religious Jews 4.4 ± 4.2); t = 2.0, *p *= 0.05).

TS resiliency was significantly associated with being male (23.6% (69/242) men vs. 5.8% (15/259) women; χ^2 ^= 32.1 (df = 1), *p *< 0.001), being Jewish (16.0% (69/430) Jews vs. 4.2% (3/71) Arabs; χ^2 ^= 6.9 (df = 1), *p *= 0.009), being born in Israel (17.6% (54/307) native Israelis vs. 8.9% (17/191) immigrants; χ^2 ^= 7.3(df = 1), *p *= 0.007), and higher income level (TS resilient 3.2 ± 1.20 vs. non TS resilient 2.7 ± 1.3; t_465 _= 2.8, *p *= 0.006).

### Regression analyses

Two logistic regressions and one linear regression were performed to assess the relative contribution of exposure to terrorist attacks, demographic items, future orientation, sense of safety, self-efficacy, life events, social support, and distress about societal problems to meeting symptom criteria of PTSD and to TS resiliency and number of TSR symptoms. The final regression models are presented in Table 6.

The three regression models showed the following significant contributions:

• Being female and being Arab contributed significantly to meeting PTSD symptom criteria, having more TSR symptoms, and not being TS resilient

• Being born in Israel contributed to TS resilience and fewer TSRS

• Optimism about the future of the State of Israel contributed to being TS resilient

• Less education contributed to more TSRS

• Sense of safety contributed to TS resilience and less TSRS

• A life-threatening experience with injury contributed to meeting PTSD criteria and having more TSR symptoms

• Greater distress about societal problems contributed to more TSRS

• Sense of social support contributed to less TSRS

• A major life event in the previous year contributed to meeting PTSD criteria

• A substantial loss of income in the past contributed to with meeting symptom criteria for PTSD and not being TS resilient.

## Discussion

### Resiliency and distress

The findings of the present survey show heterogeneous responses compared with our study 2 years previously. We found that some of the measures had improved, some worsened, and some remained unchanged, possibly due to interactive processes of habituation, stress accumulation, and the ability to compartmentalize stresses.

On the one hand, the responses on some of the measures point to an apparent reduction in distress. While around half the study participants reported feeling that their own lives (47%) and/or the lives of their friends and family were in danger (54.1%), these rates are about 13% lower than those found 2 years previously. These figures suggest that even though the ongoing terror continues to rob much of Israel's civilian population of their peace of mind and sense of safety, fewer people than previously feel significantly threatened by it. There were also substantial reductions in the percentage of respondents who reported feeling depressed (29.5% vs. 58.6%) and who reported functional impairment (15.4% vs. 22.7%).

The greater sense of safety, reduced distress, and improved functioning seem to point to a process of habituation. It cannot be ruled out, however, that the changes also stem from a reduction in the number and scope of terrorist attacks over the 2-year interval and/or from the increased visibility of preventive measures (e.g. armed guards at the entrances to pubs and restaurants) and offensive actions by the Israeli Defence Forces, which had initially adapted a more defensive stance.

Responses on some other measures, however, point to increased distress. Mean TSRS rose (5.0 vs. 4.0), TS resiliency dropped by around a third (14.4% vs. 23.3%), almost twice as many respondents reported feeling the need for professional mental-health treatment (9.7% vs. 5.3%), and fewer reported feeling optimistic about the future of the State (56.5% vs. 66.2%). This pattern of responses seems to reflect an accumulation of stress and erosion of resiliency after 4 years of ongoing terror. It highlights the need to enhance the ability of individuals and societies to withstand the psychological stress of ongoing terror, especially as terror is becoming a worldwide affliction.

Finally, responses on some measures remained unchanged. The percentage of respondents who met PTSD criteria (9%) remained roughly the same as previously (9.4%), as did their scores on the various PTSD symptom clusters. These findings are interesting, as one would expect increased rates as a result of more people being exposed over time, and the development of delayed and reactivated PTSD as a result of the repeated terror attacks.

Also unchanged were the very high percentages of respondents who reported feeling optimistic about their personal futures (82%) and feeling self-efficacy if they were caught in a terrorist attack in the future (76.6%). These findings suggest that self-confidence and abilities are not undermined by terror and may even be bolstered by it [[25], pp. 43–47] and suggesting that external threat can increase aspects of resiliency and sense of purpose [26]. At the same time, there seems to be some disparity between the respondents' optimism about their personal future and the sense of life threat, whether to themselves or to people close to them, reported by at least half the participants. This may reflect the human ability to compartmentalize.

Overall, the various patterns of responses suggest that exposure to ongoing terror may result in both habituation and erosion of resiliency. The findings point to the need to examine the long-term impact of terror, war, and other disasters on a variety of parameters [25].

As trauma studies traditionally tend to explore the pathological aspects of the consequences of trauma (e.g. PTSD, TSRS) it is important to note that resiliency is not a mere mirror reflection of PTSD. While resiliency and not PTSD is influenced by optimism, sense of safety, and immigrant status, PTSD but not resiliency is influenced by previous life-threatening experiences and major life events in the previous year.

### Societal concerns

The findings on societal concerns are also equivocal. On the one hand, two-fifths of the respondents marked either the terror attacks or the security situation as the most upsetting of the 10 societal problems listed, yet the great concern with terrorism-related issues did not prevent respondents from being very upset by more mundane social problems. Over a quarter of the respondents rated the economic situation as the most upsetting, while about a quarter to over a third reported feeling very upset about ordinary problems of civil society: corruption, lack of leadership, road accidents, and crime. These findings suggest that, for all the tension created by the ongoing terrorism, people still had energy left over for other concerns. It cannot be ruled out, however, that the tension created by terrorism did perhaps augment their distress about other matters. Further study is required to better understand this inter-relationship.

Relatively low percentages of respondents were 'very upset' about human rights issues: the treatment of Palestinians in the territories; the treatment of the country's minorities, Israeli-Arabs or foreign workers; and ethnic discrimination among Jews. It is difficult to know how much the relative unconcern stems from anger at the Palestinian terrorism and how much from a blunting of empathy and moral consciousness in the face of the survival threat posed by terrorism. Our findings are in line with the terror management theory [27] and the findings of Hobfoll et al [28], suggesting that the threat and fear of death increases prejudice, stereotyping, and derogation of outsiders.

### Risk factors

Our findings also show that various sectors of society were affected by the terror differently. In particular, being female and being Arab both contributed significantly to the likelihood of meeting symptom criteria for PTSD, to having more TSR symptoms, and not being TS resilient.

The women in our sample were four times more likely than men to meet symptom criteria for PTSD, endorsed higher levels of TSRS, and were 3.7 times less likely to be TS resilient. Their greater vulnerability is consistent with other findings [29,30]. It may be rooted in women's higher sense of threat, lower self-efficacy, and tendency to use less effective coping strategies than men [31]. It may stem from the fact that more women than men in this study had experienced a major life event in the past year. Alternatively, it may also be anchored in gender-differentiated reporting patterns, with women more ready to report distress than men.

Arabs were 2.5 times more likely than Jews to meet PTSD criteria and 5.9 times less likely to be TS resilient. These findings contrast with those of our previous study, which did not show any ethnic differences in vulnerability. They are surprising in light of the fact that Arabs were not targeted by terrorism and proportionately fewer were killed and injured in the attacks than Jews. However, these findings are consistent with those of Hobfall et al [28] and Somers et al [32], and with studies of the reactions of children to continuous stress of war and terrorism in the region [33], which have shown that the Israeli Arab minority as well as the Palestinian population has become psychologically vulnerable to terrorism.

Several non-exclusive explanations may be suggested. One is that the identification of Arab citizens of Israel with the suffering of the Palestinians in the territories, the tensions of dual allegiance, and mounting Jewish hostility in the wake of the violence may all have been sources of traumatogenic stress [34], the impact of which became increasingly apparent as the intifada raged on. Another possible explanation is their minority status in Israel. This explanation is consistent with findings showing that belonging to a minority group increases the likelihood of PTSD and trauma-related distress [35,36]. A third explanation lies in the resource deprivation of Arabs in Israel, where they earn less than Jews, are less educated, have low social status, and fewer opportunities for advancement. Their heightened vulnerability, in comparison both with that in our previous study and with the Jewish population, may stem from the depletion of their limited resources over time [28,37].

Resource deprivation may also help account for our findings that less educated respondents had more TSRS than their better educated peers, and that immigrants were less TS resilient than native-born Israelis. Although both findings are consistent with those of earlier studies [25,38], neither education nor immigration were found to be predictors of terror-induced distress in our previous study.

Consistent with our previous findings, level of exposure did not contribute to any of our measures of traumatic stress. This finding is inconsistent with findings of significant associations between level of exposure and traumatization [29]. It is, however, consistent with findings of traumatization in the wake of the '9/11' attacks among people who were very far from the areas targeted [4], as well as with findings in Israel showing that level of exposure was not necessarily related to symptom severity [15,39,40]. Thus, when terror assumes a national dimension, people do not have to experience it first-hand to suffer its psychological consequences. An explanation may lie in the process of social/cultural distress reappraisal or calibration [37] that accompanies a national threat of this amplitude.

In contrast to current exposure to terror, however, previous exposure to trauma or stress (e.g. prior traumatic experience, substantial income loss, and a highly stressful or emotional experience in the previous year) did contribute to vulnerability in the face of terror, while distress over societal problems contributed to increased TSRS.

### Limitations

When evaluating our conclusions, it should be noted that although our questionnaires included validated, widely used questionnaires, we added study-specific questions, thus not all items in our evaluation instruments had been previously validated.

The limitations include the lack of data from before the Intifada on the psychological repercussions examined in this study. We also cannot know whether the refusal of those who declined the interview was associated with higher levels of distress. Furthermore, we cannot determine whether self-reported symptoms are clinically significant or merely reflect heightened awareness and agitation due to the threat of terrorism. Caution should be taken when generalizing findings to various subpopulations that may or may not have been exposed to the threat of terrorism and may not have been adequately represented in the strata sampling (e.g. those without homes or telephones).

However, the large sample size, high response rate, and lack of significant demographic differences between participants and those who refused to participate support the credibility of our findings.

## Conclusion

This study shows that Israeli society has coped with nearly 4 years of intense and continuous terror in a mixed manner, and suggests that, aside from possibly fostering habituation, continuous terror results in erosion of resiliency. The findings also show that the erosion of resiliency disproportionately affects groups with fewer basic resources, including the Arab population, the less educated, and immigrants. Finally, our findings suggest that known vulnerability factors such as gender and exposure to previous traumatic events contribute to the prediction of terrorism-related distress.

## Competing interests

The author(s) declare that they have no competing interests.

## Authors' contributions

AB, MG and ZS were responsible for the study concept and design. MG was responsible for acquisition of data, and MG, AB, and ZS for analysis and interpretation of data. The drafting of the manuscript was carried out by MG and AB, and critical revision of the manuscript for important intellectual content by AB, MG, YM and ZS. Statistical expertise was provided by MG, and study supervision carried out by AB, MG, YM and ZS. MG, AB, YM and ZS had full access to all of the data in the study and take responsibility for the integrity of the data and the accuracy of the data analysis.

## Pre-publication history

The pre-publication history for this paper can be accessed here:



## Figures and Tables

**Table 1:** Demographic characteristics of survey participants (*n *= 501).

Characteristics	Number (%)
Age (years), mean ± SD (range)	44.81 ± 17.1 (18–91)
Gender	
Male	242 (48.3)
Female	259 (51.7)
Ethnicity	
Jews	430 (85.8)
Israeli Arabs	71(14.2)
Education	
Elementary school only	29 (5.8)
High school	220 (43.9)
Higher education	252 (50.3)
Religiosity (Jews, *n *= 426)*	
Orthodox	25 (5.9)
Religious	39 (9.2)
Traditional	122 (28.6)
Atheist	240 (56.3)
Residence	
Urban	382 (76.2)
Agricultural, village communities (including kibbutzim)	35 (7.0)
Arab villages	71 (14.2)
Settlements outside 1967 border	13 (2.6)
Place of birth: (Jews; *n *= 430)†	
Israel	241 (56.0)
Outside of Israel	191 (44.0)
Income	
Below mean	189 (40.5)
Mean (US $2000)	132 (28.3)
Above mean	146 (31.2)

*Religiosity data were not obtained for Arab participants.

†All Arab participants were born in Israel.

**Table 2:** Rate of exposure to terrorism and other life events, life threatening experiences, loss of income, and ethnic discrimination

	No.	%
No personal exposure and no exposure of friends or relative	344	68.7
No personal exposure but exposure of friends or relative who were not wounded	35	7.0
No personal exposure but exposure of friends or relative who were wounded	34	6.8
No personal exposure but exposure of friends or relative who died	32	6.4
Personal exposure: no physical wounds, but no exposure of friends or relative	25	5.0
Personal exposure: no physical wounds, and exposure of friends or relative who were not wounded	10	2.0
Personal exposure: no physical wounds, and exposure of friends or relative who were wounded	8	1.6
Personal exposure: no physical wounds, and exposure of friends or relative who died	11	2.2
Personal exposure with physical wounds but no exposure of friends or relative	2	0.4
Lifetime life-threatening experience*	179	35.9
Lifetime life-threatening experience with injury	36	7.2
Substantial loss of income	175	35.0
Victim of ethnic discrimination	70	14.0
Major life event in previous year	211	42.3

**Table 3:** Frequency of symptom criteria for PTSD.

	May 2004 study (*n *= 501)		April 2002 study (*n *= 512)	
	
Symptoms	Symptoms endorsed, mean ± SD (range)	Respondents with ≥ 1 symptom, no. (%)	Symptoms endorsed, mean ± SD (range)	Respondents with ≥ symptom, no. (%)
Re-experiencing (B) cluster	0.6 ± 0.9 (0–4)	180 (35.9)	0.6 ± 0.9) (0–4)	189 (37.1)
Avoidance/numbing (C) cluster	1.0 ± 1.3 (0–6)	259 (51.7)	1.1 ± 1.4 (0–6)	283 (55.5)
Hyperarousal (D) cluster	1.2 ± 1.6 (0–6)	239 (47.7)	1.1 ± 1.5 (0–6)	252 (49.4)
Functional impairment	0.2 ± 0.4 (0–2)	77 (15.4)	0.3 ± 0.5(0–2)	116 (22.8)
General distress	0.4 ± 0.5 (0–1)	221 (44.1)	0.5 ± 0.5 (0–1)	236 (46.3)
Dissociative cluster	0.4 ± 0.8 (0–4)	136 (27.1)	0.4 ± 0.8 (0–4)	138 (26.9)
Total number of TSRS symptoms	5.0 ± 4.5 (0–23)	429 (85.6)	4.0 ± 4.5 (0–23)	391 (76.7)
Met symptom criteria for PTSD	*n *= 45 (9%)		*n *= 48 (9.4%)	
TS resiliency	*n *= 72 (14.4%)		*n *= 121 (23.3%)	

**Table 4:** Percentage of participants reporting each trauma related feelings and behaviors (*n *= 501).

	Endorsements, *n *(%)	
Items	April 2004 study, *n *= 501	May 2002 study, *n *= 512

Depression	147 (29.5)	299 (58.6)
Tension and anxiety	145 (29.0)	N/A
Optimism about personal future	409 (82.0)	421 (82.2)
Optimism about the future of Israel	279 (56.5)	337 (66.2)
Low sense of personal safety	235 (47.0)	307 (60.4)
Low sense of safety of friends and relatives	270 (54.1)	345 (67.9)
Self-efficacy in terror attack	308 (76.6)	322 (74.6)
Sense of social support	390 (78.2)	N/A
Need mental health treatment*	48 (9.7)	27 (5.3%)

N/A, not applicable.

All items were endorsed at 'agree' (third choice of 5) or higher, except for those marked with *; percentages on these items reflect a positive answer to yes/no questions.

**Table 5:** Distress about societal issues

Distress about:	Very distressed,* *n *(%)	Most distressing,† *n *(%)
The economic situation	133/499 (26.7)	126 (26.1)
The terror attacks	121/500 (24.2)	118 (24.4)
Security situation	141/499 (28.3)	73 (15.1)
Administration corruption	180/498 (36.1)	50 (10.4)
Lack of leadership	143/492 (29.1)	46 (9.5)
Violence and crime	136/498 (27.3)	24 (5.0)
Treatment of Palestinians in the territories	82/482 (17.0)	17 (3.5)
Motor vehicle accidents	158/500 (31.6)	14 (2.9)
Ethnic discrimination	27/497 (5.4)	12 (2.5)
Treatment of minorities (Arabs, Bedouins, foreign workers)	94/495 (19.0)	3 (0.6)

*Items endorsed 'very much' (fifth choice of 5).

†Forced-choice items.

**Table 6:** Summary of hierarchical regression analysis (TSR symptoms) and logistic regression analysis (PTSD symptoms and TS resiliency)

Variable	PTSD symptoms, OR (95% CI)	TSR symptoms, standard B ± SE	Not TS resilient, OR (95% CI)
Female	4.0 (1.8–8.6)	-0.18 ± 0.35	3.7 (1.9–7.4)
Arab (vs. Jewish) ethnicity	2.5 (1.2–5.5)	-0.15 ± 0.52	5.9 (1.6–21.2)
Born outside of Israel	N/A	-0.11 ± 0.38	2.8 (1.5–5.5)
Low optimism about state	N/A	N/A	1.4 (1.1–1.7)
Less education	N/A	0.11 ± 0.05	N/A
Low sense of safety	N/A	0.19 ± 0.09	1.8 (1.3–2.4)
Life-threatening experience with injury in the past	4.5 (1.7–12.1)	0.15 ± 0.64	N/A
Higher distress over societal problems	N/A	-0.18 ± 0.02	N/A
Lower sense of social support	N/A	0.08 ± 0.12	N/A
Major life events in previous year	2.0 (1.0–3.9)	N/A	N/A
Substantial loss of income in the past	3.1 (1.6–6.1)	N/A	2.4 (1.2–5.0)

N/A, not applicable; TS, traumatic stress. TSRS, traumatic stress-related symptoms.

Total R^2 ^for variables predicting TSR symptoms = 38.5.

Respondents meeting symptom criteria for PTSD = 45/501 (9%); TS-resilient respondents = 71/501 (14.4%).

*p *< 0.05 for all variables.
